# Transforaminal Epidural Steroid Injection in Lumbar Disc Prolapse: Impact on Pain Intensity and Cognitive Function in Relation to MicroRNA-155 Serum Level

**DOI:** 10.1155/anrp/2201031

**Published:** 2025-04-07

**Authors:** Wael Fathy, Mona Hussein, Rehab Magdy, Mona Nasser, Jehan Mohamed, Doaa Moaz Sayem, Hatem Elmoutaz, Nesma Mounir, Dina Mahmoud Fakhry, Mohamed Abdelbadie

**Affiliations:** ^1^Department of Anesthesiology, Surgical Intensive Care and Pain Management, Beni-Suef University, Beni-Suef, Egypt; ^2^Department of Neurology, Beni-Suef University, Beni-Suef, Egypt; ^3^Department of Neurology, Cairo University, Giza, Egypt; ^4^Department of Clinical & Chemical Pathology, Beni-Suef University, Beni-Suef, Egypt

**Keywords:** cognitive function, lumbar disc prolapse, miRNA-155, pain and disability scales, TFESI

## Abstract

**Background:** Lumbar disc prolapse is a common cause of disabling low back pain. The prevalence of disc prolapses or herniation in the general population increases with age.

**Objective:** This work aimed to evaluate the impact of transforaminal epidural steroid injection (TFESI) in lumbar disc prolapse on pain intensity, cognitive function, and miR-155 serum level.

**Methods:** The present case-control study was conducted on 44 patients with symptomatic lumbar disc prolapse (L4-L5) and another 44 age- and sex-matched controls. Assessment of the pain intensity and functional disability was done before and 1 month after TFESI using the numeric rating scale (NRS), Oswestry disability index (ODI), and functional rating index (FRI). Cognitive assessment was done before and 1 month after TFESI. Estimation of miR-155 serum level was done for the included patients (before and 1 month after TFESI) and controls.

**Results:** There was a statistically significant improvement in pain scales and cognitive test scores 1 month following TFESI (*p* value ≤ 0.05 in all comparisons). There was also a statistically significant reduction in miRNA-155 serum level in the included patients one month following TFESI (*p* value < 0.001). The median values for the change in NRS were 2 (1–4.75), in ODI were 18 (7–33), in FRI were 23.5 (12–31), in PALT were 1 (0–1.5), in COWAT were 2 (0.25–5), in PASAT were 3 (1.25–4), and in miRNA-155 were 0.555 (0.16–0.738). There were statistically significant correlations between miRNA-155 serum levels in the included patients and the scores of all the pain and disability scales (NRS, ODI, and FRI) and the scores of all the cognitive tests before TFESI (*p* value ≤ 0.05 in all correlations).

**Conclusion:** This study highlights the epigenetic mechanisms of TFESI in lumbar disc prolapse, causing significant downregulation of miRNA-155, reduced pain intensity, and improved cognitive function.

**Trial Registration:** ClinicalTrials.gov identifier: NCT05626283

## 1. Background

Lumbar disc prolapse is a common cause of disabling low back pain, with a peak incidence in middle age (30–50 years) [1]. The underlying pain mechanism is related to a complex interaction between inflammatory, compressive, and immunological factors. The pressure exerted by the prolapsed disc on the longitudinal ligaments and the irritation caused by the released inflammatory chemokines result in localized back pain. The lumbar disc-related radicular pain arises when the prolapsed disc exerts pressure on the thecal sac or lumbar nerve roots, resulting in nerve root inflammation and ischemia [2].

Chronic pain has been associated with poor cognitive functions in multiple domains, especially in the older population. Significant cognitive dysfunction has been reported in the domains of memory, executive functioning, attention abilities, psychomotor, and processing speeds [3]. Also, chronic pain was associated with physical limitations in walking speed and activities of daily living [4].

MicroRNAs (miRNAs) are small endogenous RNAs that repress gene expressions post-transcriptionally. They are involved in the processes of neuropathic pain regulation through targeting the 3′UTR of mRNAs [5]. Multiple miRNAs have been studied as regulators of neuropathic pain through involvement in nociceptive pathways, such as miRNA-155, miRNA-21, and miRNA-146 [6]. Specifically, miR-155 is a multifunctional miRNA, a fundamental regulatory factor in immune response, neuroinflammation, cognitive impairment, and neuropathies [7]. It was also noticed to be dysregulated in cases of intervertebral disc degeneration and promoting annulus fibrosis catabolism and related neuropathic pain [8]. Previous studies suggest evidence that miRNA-155 promotes the release of pro-inflammatory cytokines, nuclear factor-κB, and mitogen-activated protein kinase activation (MAPK) signaling [9]. miR-155 also has a role in inflammatory reactions and targeting inflammation-related proteins such as serum and glucocorticoid-regulated protein kinase 3 (SGK3) [10].

Transforaminal epidural steroid injection (TFESI) is a common intervention for the treatment of neuropathic pain related to disc prolapse. Steroids inhibit the production of several proinflammatory mediators, reducing nerve root inflammation induced by mechanical compression. Further, it can block neural transmission through nociceptive nerve fibers [11, 12].

A paucity of data are available about the effect of the different therapeutic modalities of lumbar disc-related radicular or low back pain on the pain-related functional and structural brain alterations and the associated cognitive dysfunction [13].

Taken together, we hypothesized that the altered regulation of miR-155 might contribute to improved pain and cognitive dysfunctions in patients with lumbar disc prolapse undergoing TFESI.

## 2. Methods

### 2.1. Study Design and Participants

In this case-control study, 44 patients diagnosed with symptomatic lumbar disc prolapse (L4-L5) (case group) and another 44 healthy volunteers (control group) as a reference were evaluated. Both groups were age- and sex-matched. Patients were recruited from the neurology and pain management outpatient clinics at the Beni-Suef Pain Center during the period from December 2022 to September 2024. The healthy controls were recruited from the patients' relatives. They should be free from any medical illness known to cause pain or impact cognition.

**Figure 1:**
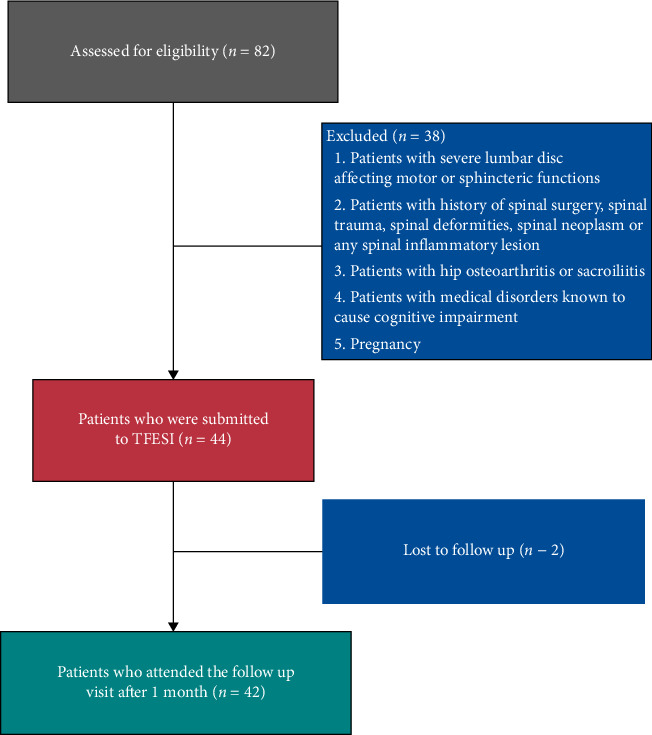
Flow diagram for the included and excluded patients. TFESI: transforaminal epidural steroid injection.

This study included patients with clinical and radiological evidence of L4-L5 disc prolapse manifesting as radicular pain and low back pain unresponsive to medical treatment and physiotherapy over a period of at least 3 months. Exclusion criteria included patients with severe lumbar disc prolapse affecting motor or sphincteric functions, history of spinal surgery, spinal trauma, spinal deformities, spinal neoplasm, any spinal inflammatory lesion, hip osteoarthritis, or sacroiliitis. Also, patients with medical or psychiatric disorders based on DSM-V [14], known to cause cognitive impairment and pregnant women were excluded. The flow diagram for the included and excluded patients is illustrated in Figure 1.

The study was conducted in two stages. In the first stage, baseline serum levels of miRNA-155 and cognitive assessments were performed for both case and control groups. Further, at this stage, the case group was evaluated in terms of pain intensity and functional disability. Demographic data and the duration of lumbar disc-related radicular pain were obtained.

The second stage was carried out one month after TFESI, in which the case group (*n* = 44) with symptomatic lumbar disc prolapse was evaluated regarding miRNA-155 serum level, pain intensity, functional disability, and cognitive testing to explore the effect of steroid injection on these variables. A 1-month post-TFESI interval was chosen because this timeline aligns with the pharmacodynamic effects of the steroids, so a 1-month follow-up was used in clinical trials evaluating the short-term efficacy of TFESI [15, 16].

### 2.2. Pain Intensity and Functional Disability

The following scales were used for the assessment of pain intensity and functional disability as described by a prior clinical trial [17].

#### 2.2.1. Numeric Rating Scale (NRS) [18]

It is an 11-point numeric scale for radicular pain intensity evaluation, ranging from 0 to 10, where higher scores denote more intense pain.

#### 2.2.2. Oswestry Disability Index (ODI) [19]

It is a scale used to measure functional incapacity from low back pain across many aspects, including lifting, walking, standing, sitting, personal care, sleeping, traveling, work, and social life. It consists of 10 questions, scored on a scale of 0–5 for each, where higher scores denote more limitations. After summating all these subscores, the result is multiplied by two to gain the index (ranging from 0 to 100).

#### 2.2.3. Functional Rating Index (FRI) [20]

Another scale was used to weigh functional limitations in daily activities in people with low back pain. The scale consists of 10 items scored 0–4 for each, where higher scores denote more disability.

### 2.3. Cognitive Assessment

The cognitive tests were performed by a trained neuropsychologist who was blind to group allocation and unaware of the study's purpose to minimize bias and maximize the validity of the results. Some investigators may be eager to see a positive outcome after the procedure.

#### 2.3.1. Paired Associate Learning Test (PALT) [21]

This test was used to assess episodic verbal memory. Six semantically related word pairs and four unrelated pairs were introduced to the subject. After that, the patient was requested to retrieve the word paired with each. The test was conducted on three trials. A score of 0.5 is given for each true, well-matched pair, while a score of 1 is given for each true, mismatched pair, with a total score ranging between 0 and 21.

#### 2.3.2. Paced Auditory Serial Addition Test (PASAT) [22]

This test was used to assess attention and processing speed. A set of single digits is audio recorded at a rate of one every 3 s and then introduced to the subject. The subject was requested to add each digit to the one just heard before. The number of correct answers was summed to gain the total score (0–60).

#### 2.3.3. Controlled Oral Word Association Test (COWAT) [23]

It is a verbal fluency test that assesses executive function. The subject was asked to generate words beginning with a specified letter in 1 min, eliminating proper nouns.

### 2.4. Interventional Pain Procedure

In the preparation room, the patient was placed in a prone position on a fluoroscopy table and draped in a sterile manner. The patient was connected to a monitor (BP, SPO2, and ECG) and given supplemental oxygen through a nasal cannula (3 L/min). Under fluoroscopic guidance, a spinal needle (a 22-gauge, 3.5-inch) was advanced in an oblique view. The classic Scottie dog view was obtained to access the safe triangle, and the needle was directed toward the inferior-lateral boundary of the pedicle. Both lateral and anterior-posterior views were obtained to confirm the appropriate location of the needle between L4-L5. With each insertion of the spinal needle, 1 mL with 20 mg of local anesthetic lidocaine 2% was injected intradermally. The TFESI with local anesthetic (triamcinolone acetonide 80 mg [2 mL] and bupivacaine hydrochloride 0.5% [3 mL]).

### 2.5. Laboratory Assessment

Real-time PCR evaluation of miRNA-155 by quantitative reverse transcription (qRT).

### 2.6. Sample Collection

One venous blood sample was collected from each of the 44 healthy controls, while two samples were obtained from each of the 44 patients with lumbar disc prolapse: a baseline sample was taken first, followed by a second one a month after the steroid injection.

### 2.7. Extraction, Purification, and Transcription

According to the manufacturer's protocol, serum RNA was extracted using the miRNeasy kit (catalogue number 217184, Qiagen). Transcription of miRNA into complementary DNA (cDNA) by the miScript II RT kit (catalogue number 218161, Qiagen) was performed under the following conditions: incubation of 20 μL reverse transcription reaction for 60 min at 37°C, followed by 5 min at 95°C to stop the reverse transcriptase. 200 μL of RNase-free water was used to dilute cDNA. Complementary DNA was stored at −80°C till PCR processing.

### 2.8. Real-Time PCR Detection and Relative Quantification (RQ)

In the qRT-PCR assay, specific forward primers with Qiagen catalogue numbers (MS00031486 for microRNA 155 and MS00033712 for SNORD-68 [endogenous control]) were used along with the Qiagen reverse universal primer and the Syber Green PCR Master Mix according to the manufacturer's instructions.

Real-time PCR thermal cycles included an initial non-repetitive activation step at 95°C for 15 min followed by 40 repeated cycles. Each cycle comprised 3 main steps: denaturation at 94°C for 15 s, annealing at 55°C for 30 s, in addition to extension for 30 s at 70°C. RQ of miRNA 155 was performed by using the 2^−ΔΔCt^ equation (comparative method, or ΔΔCT method) [24]. Normalization was achieved by using SNORD 68 as an internal control [24].

### 2.9. Outcomes of the Study

The primary outcome of this work was the comparison of miRNA-155 before and one month after TFESI in patients with lumbar disc prolapse to clarify the possible changes that may occur in miRNA-155 serum level in patients with lumbar disc prolapse 1 month after TFESI.

The secondary outcome was to study pain intensity and cognitive function before and 1 month after TFESI. Also the relationship between pain intensity, cognitive function, and miRNA-155 serum level in patients with lumbar disc prolapse before and one month after TFESI.

### 2.10. Sample Size Calculation

Sample size calculation was done using the comparison of miRNA before and after TFESI in patients with lumbar disc prolapse. Searching the literature failed to find any previous results that can be used to build up sample size. Therefore, we performed a pilot study to get usable results. As reported in our pilot study, the mean ± SD of miRNA before the injection was 1.56 ± 0.178, while after the injection, it was 0.912 ± 0.146. Accordingly, we calculated that the minimum proper sample size was 27 participants to be able to detect a real difference of 0.1 units with 80% power at the *α* = 0.05 level using a paired *t-*test for dependent samples. Sample size calculation was done using Stats Direct statistical software version 2.7.2 for MS Windows, StatsDirect Ltd., Cheshire, UK.

### 2.11. Statistical Analysis

IBM Statistical Package of Social Science (SPSS) Version 25 was used to analyze the data. The Kolmogorov–Smirnov test was used to test the normality of data. Categorical variables such as sex, discogenic pain, and radicular pain were expressed as numbers and percentages. Non-normally distributed quantitative variables, such as duration of pain, pain disability scales, cognitive function tests, and miRNA-155 serum level, were expressed as the median and interquartile range (IQR). Normally distributed quantitative variables, such as age, were expressed as mean and standard deviation. Chi-squared test was used for comparison between patients and controls in categorical variables. An independent sample *t*-test was used for comparison between patients and controls in age. Wilcoxon test was used for comparison between pain disability scales, cognitive function tests, and miRNA-155 serum levels in the included patients before and after intervention. Correlations between pain disability scales, cognitive function tests, and miRNA-155 serum levels were done using the Spearman correlation test. *p* value ≤ 0.05 was considered statistically significant. All tests were two-tailed.

## 3. Results

Demographics, clinical and radiological characteristics, and miRNA-155 serum levels in the included patients in comparison to controls.

The present case-control study was conducted on 44 patients diagnosed with lumbar disc prolapse and 44 age- and sex-matched healthy controls (*p* value = 0.838 and 0.286, respectively). Regarding the clinical characteristics of the included patients, the median value for disease duration was 24 (7.25–36) months. Only 16 (36.4%) patients had discogenic pain, 24 (54.5%) patients had unilateral radicular pain, and 20 (45.5%) patients had bilateral radicular pain.

Assessment of pain and disability revealed that the median value for NRS score before TFESI was 6.5 (5–8), for ODI score was 58 (50–66), and for FRI was 75 (47.75–82) (Table 1).

Regarding cognitive function before TFESI, there were statistically significant differences between patients and controls in the scores of PALT, COWAT, and PASAT (*p* value = 0.005, 0.002, and 0.045, respectively) (Table 1).

The included patients were found to have a significantly higher median value of miRNA-155 serum level before TFESI (1.48 [0.94–1.64]) in comparison to controls (0.83 [0.72–0.93]) (*p* value < 0.001) (Table 1).

### 3.1. The Impact of TFESI on Pain and Disability, Cognitive Function, and miRNA-155 Serum Level in Patients Having Lumbar Disc Prolapse

Two patients did not attend the follow-up visit, so the total number of patients analyzed was 42 (Figure 1). Regarding pain and disability scales, there was a statistically significant improvement in the scores of NRS, ODI, and FRI 1 month following TFESI (*p* value < 0.001 in all comparisons) (Table 2).

As for cognitive function tests, there was also a statistically significant improvement in the scores of PALT, COWAT, and PASAT 1 month following TFESI (*p* value = 0.019, < 0.001, < 0.001, respectively) (Table 2).

Additionally, there was a statistically significant reduction in miRNA-155 serum level in the included patients one month following TFESI (*p* value < 0.001) (Table 2).

The median values for the change in NRS were 2 (1–4.75), in ODI were 18 (7–33), in FRI were 23.5 (12–31), in PALT were 1 (0–1.5), in COWAT were 2 (0.25–5), in PASAT were 3 (1.25–4), and in miRNA were 155 0.555 (0.16–0.738).

### 3.2. The Relationship Between Pain and Disability and Cognitive Function Before and One Month After Intervention

There were statistically significant negative correlations between the scores of all the pain and disability scales in the included patients (NRS, ODI, and FRI) and the scores of all the cognitive function tests (PALT, COWAT, and PASAT) before TFESI (*p* value ≤ 0.05 in all correlations) (Table 3).

On the other hand, there were no statistically significant correlations between the scores of any of the pain and disability scales (NRS, ODI, or FRI) and the scores of any of the cognitive function tests (PALT, COWAT, or PASAT) after TFESI (*p* value > 0.05 in all correlations) (Table 3).

### 3.3. The Relationship Between miRNA-155 Serum Level and Pain and Disability Before and One Month After Intervention

There were statistically significant positive correlations between miRNA-155 serum levels in the included patients and the scores of all the pain and disability scales (NRS, ODI, and FRI) before TFESI (*p* value ≤ 0.05 in all correlations) (Table 4).

On the other hand, there were no statistically significant correlations between miRNA-155 serum level and the scores of any of the pain and disability scales (NRS, ODI, or FRI) after TFESI (*p* value > 0.05 in all correlations) (Table 4).

### 3.4. The Relationship Between miRNA-155 Serum Level and Cognitive Function Before and One Month After Intervention

There were statistically significant negative correlations between miRNA-155 serum levels in the included patients and the scores of all the cognitive function tests (PALT, COWAT, and PASAT) before TFESI (*p* value ≤ 0.05 in all correlations) (Table 4).

On the other hand, there were no statistically significant correlations between miRNA-155 serum level and the scores of any of the cognitive function tests (PALT, COWAT, or PASAT) after TFESI (*p* value > 0.05 in all correlations) (Table 4).

### 3.5. The Relationship Between miRNA-155 Levels Pre- and Post-TFESI

**Figure 2:**
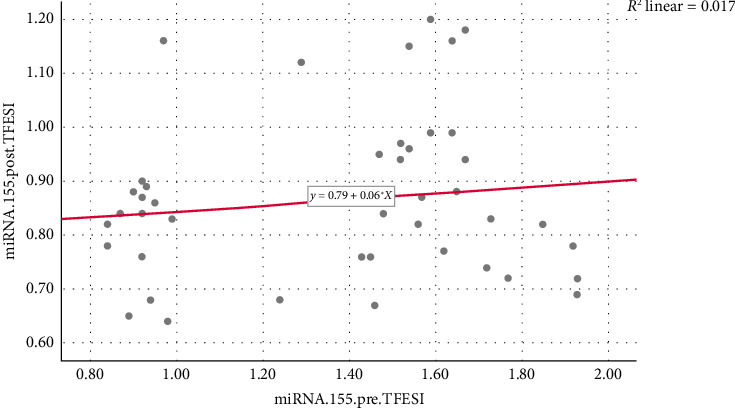
Correlation between miRNA-155 levels pre- and post-TFESI. TFESI: transforaminal epidural steroid injection.

There was no statistically significant correlation between miRNA-155 levels pre- and post-TFESI (*r* coef. = 0.104, *p* value = 0.503). A scatter plot was designed to demonstrate the relationship between miRNA-155 levels pre- and post-TFESI (Figure 2).

The interplay between miRNA-155, pain intensity, and cognitive scores:

  Before TFESI: miRNA-155 levels are positively correlated with pain intensity (NRS) and negatively correlated with cognitive scores.
  After TFESI: Correlations weaken or shift, showing minimal relationships post-intervention.

**Figure 3:**
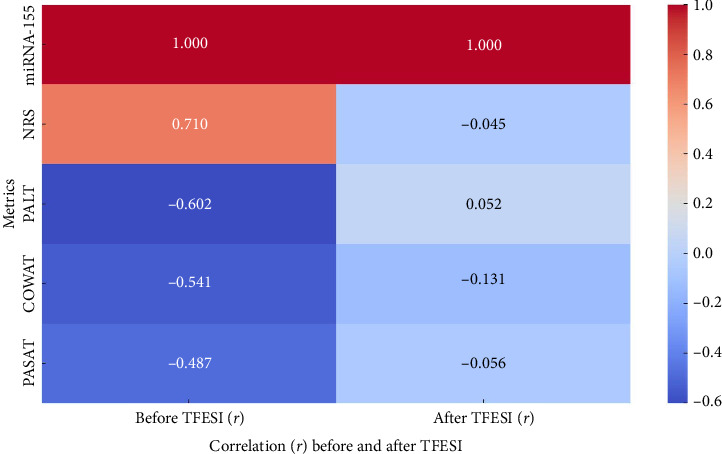
Interplay between miRNA-155, pain intensity, and cognitive scores. NRS: numeric rating scale, PALT: paired associate learning test, COWAT: controlled oral word association test, PASAT: paced auditory serial addition test, TFESI: transforaminal epidural steroid injection, and (*r*) coef.: Pearson correlation coefficient.

The visualization highlights how the interplay between these factors changes with treatment (Figure 3).

## 4. Discussion

Studying epigenetic alteration induced by pain therapeutic interventions provides new insights into pathways involved in the therapeutic efficacy of such modalities. The present study confirmed favorable short-term outcomes of TFESI in patients with lumbar disc prolapse, including pain reduction, cognitive improvement, and downregulation of miRNA-155.

Our data showed that miR-155 was overexpressed in lumbar disc prolapse patients more than in healthy controls, suggesting that miR-155 might participate in the pathological mechanism of radicular pain. The role of miR-155 has been previously acknowledged in experimental models of neuropathic pain [10, 25, 26], in which miR-155 was overexpressed in neuropathic pain rats, and the pain could be alleviated by miR-155 suppression.

The miR-155 exerted its pro-inflammatory response via various pathways. First, miR-155 is a dynamic regulator of Toll-like receptor (TLR) 3 and 4 and interferon (IFN)-γ-mediated signaling through the nuclear factor κB (NF-κB) [9, 27]. Second, miR-155 is highly upregulated in activated microglia and endorses the release of pro-inflammatory cytokines such as IL-1β and TNFα [9, 28]. All these pro-inflammatory mediators are principally involved in the inflammatory-driven mechanism of neuropathic pain in disc prolapse [29]. Moreover, miR-155 induces neuroinflammation through the inhibition of anti-inflammatory target genes such as Suppressor of Cytokine Signaling (SOCS1), a negative regulator of cytokines [30], and SH2 Domain-Containing Inositol 5′-Phosphatase1 (SHIP1), a negative regulator of TNF-α [31].

Interestingly, miR-155 inhibitors could suppress the expression of pro-inflammatory cytokines, including IL-1β, IL-6, and TNF-α [32, 33]. Strong former evidence proved that various mechanisms could be attributed to the anti-inflammatory effect of TFESI, and the reducing effect on such pro-inflammatory cytokines is one of these evident mechanisms [34, 35]. Therefore, based on the results presented in this study, downregulated miR-155 in patients undergoing TFESI may account for an epigenetic modulating mechanism contributing to the low-level production of TNF-α and IL-1β. Taken together, miR-155 inhibitors may constitute a potential therapeutic approach complementing TFESI in lumbar disc prolapse.

In chronic pain disorders, pain intensity, and cognitive dysfunction are united by neuroinflammation as a primary contributing mechanism of each [36]. This might explain the significant correlations between scores of cognitive tests, pain assessment scales, and miR-155 levels determined by this study. Prior observations indicate that the degree of cognitive deficits is correlated with pain intensity in people with chronic low back pain [37–39]. On the other hand, miR-155 was studied in relation to cognitive performance in various vivo models of cognitive deficits. It was found that miR-155 inhibitors could restore cognitive performance in rat models of Alzheimer's disease by mitigating the upregulation of apoptotic Caspase-3 in the hippocampus [40] as well as experimental traumatic brain injury in mice [41].

Astoundingly, correlations between clinical assessment scales and miR-155 levels in this study turned non-significant one month after the procedure. This might indicate that the observed improvement of TFESI on each of these factors was not equivalent or a dissociation between miRNA-155 and pain and cognitive recovery timelines. Some factors might improve more than others, which could explain such convey. On the other hand, a multimodal approach targeting other molecular pathways might lead to better outcomes.

The present study has some limitations that are worthy of mention. The first limitation is the short-term treatment response to TFESIs. Longitudinal studies are needed to assess the sustainability of TFESI effects on pain, cognition, and miRNA-155i. Secondly, studying a single miRNA may not be sufficient to understand the precise role of miRNAs in chronic pain pathogenesis. Investigating a network of miRNAs rather than a single marker will likely provide a holistic view of molecular changes in chronic pain pathophysiology. Thirdly, the reliability of the self-reporting pain assessment scales used in this study might be affected by the patient's physiological and psychological status, as well as the individual difference in nociceptive perception. So, objective pain assessment tools such as functional magnetic resonance imaging (fMRI), MR spectroscopy (MRS), and electroencephalography (EEG) are recommended to obtain more reliable results. Also, the relatively small sample size, potential biases in patient recruitment (e.g., control group from patients' relatives), and variability in qPCR results were worthy of note. Finally, it should be noted that a few of the included patients might be regarded as chronic pain patients due to their high mean age and long pretreatment duration. Whether or not this positive effect on miR-155 would also extend to chronic pain cases is a question that future studies will answer.

## 5. Conclusion

This study highlights the epigenetic mechanisms of TFESI in lumbar disc prolapse, causing significant downregulation of miRNA-155, reduced pain intensity, and improved cognitive function. Future studies will test the therapeutic potential of miRNA-155 inhibition, complementing TFESI, for reducing low back pain in lumbar disc prolapse.

## Figures and Tables

**Table 1:** Demographics, clinical and radiological characteristics, and microRNA-155 serum level in the included patients (case group) in comparison to healthy volunteers (control group).

		(Case group) (*n* = 44)	(Control group) (*n* = 44)	*p* value
Age (mean [SD])		49.75 (10.94)	49.27 (10.94)	0.838

Sex (*n* [%])	Males	24 (55.8%)	19 (43.2%)	0.286
	Females	20 (45.5%)	25 (56.8%)	

Duration of pain in months (median [IQR])		24 (7.25–36)		

Pain and disability scales before TFESI (median [IQR])	NRS	6.5 (5–8)	—	
	ODI	58 (50–66)	—	
	FRI	75 (47.75–82)	—	

Cognitive function tests before TFESI (median [IQR])	PALT	12.25 (10.13–15.38)	15 (13–16)	0.005⁣^∗^
	COWAT	17.50 (12–22)	21 (19–23)	0.002⁣^∗^
	PASAT	35.50 (28.5–48.75)	44 (36.25–49.75)	0.045⁣^∗^

MicroRNA-155 serum level before intervention (median [IQR])		1.48 (0.94–1.64)	0.83 (0.72–0.93)	< 0.001⁣^∗^

*Note:p* value ≤ 0.05 is considered significant.

Abbreviations: COWAT = controlled oral word association test, FRI = functional rating index, NRS = numeric rating scale, ODI = Oswestry disability index, PALT = paired associate learning test, PASAT = paced auditory serial addition test, TFESI = transforaminal epidural steroid injection.

**Table 2:** Pain and disability scales, cognitive function tests, and microRNA-155 serum level in the case group before and 1 month after TFESI.

		Before TFESI	1 month after TFESI	*p* value
Pain and disability scales (median [IQR])	NRS	6.5 (5–8)	4 (3–5)	< 0.001⁣^∗^
	ODI	58 (50–66)	33 (28–48)	< 0.001⁣^∗^
	FRI	75 (47.75–82)	41.50 (35–52)	< 0.001⁣^∗^

Cognitive function tests (median [IQR])	PALT	12.25 (10.13–15.38)	14 (11–16)	0.019⁣^∗^
	COWAT	17.50 (12–22)	21.50 (14.25–24.75)	< 0.001⁣^∗^
	PASAT	35.50 (28.5–48.75)	42.50 (32–51.50)	< 0.001⁣^∗^

MicroRNA-155 serum level (median [IQR])		1.48 (0.94–1.64)	0.84 (0.76–0.95)	< 0.001⁣^∗^

*Note:p* value ≤ 0.05 is considered significant.

Abbreviations: COWAT = controlled oral word association test, FRI = functional rating index, NRS = numeric rating scale, ODI = Oswestry disability index, PALT = paired associate learning test, PASAT = paced auditory serial addition test, TFESI = transforaminal epidural steroid injection.

**Table 3:** Correlations between pain and disability scales, and cognitive function tests in the case group before and 1 month after TFESI.

Cognitive function tests before intervention	NRS before TFESI		ODI before TFESI		FRI before TFESI	
	(*r*) coef.	*p* value	(*r*) coef.	*p* value	(*r*) coef.	*p* value
PALT before intervention	−0.618	< 0.001⁣^∗^	−0.452	0.002⁣^∗^	−0.532	< 0.001⁣^∗^
COWAT before intervention	−0.738	< 0.001⁣^∗^	−0.506	< 0.001⁣^∗^	−0.459	0.002⁣^∗^
PASAT before intervention	−0.675	< 0.001⁣^∗^	−0.624	< 0.001⁣^∗^	−0.528	< 0.001⁣^∗^

**Cognitive function tests after intervention**	**NRS after TFESI**		**ODI after TFESI**		**FRI after TFESI**	
	**(*r*) coef.**	**p** **value**	**(*r*) coef.**	**p** **value**	**(*r*) coef.**	**p** **value**

PALT after intervention	−0.121	0.432	0.172	0.263	−0.149	0.336
COWAT after intervention	−0.152	0.323	0.112	0.471	−0.172	0.263
PASAT after intervention	−0.126	0.416	0.150	0.330	−0.239	0.119

*Note:* (*r*) Coef.: Pearson correlation coefficient, *p* value ≤ 0.05 is considered significant.

Abbreviations: COWAT = controlled oral word association test, FRI = functional rating index, NRS = numeric rating scale, ODI = Oswestry disability index, PALT = paired associate learning test, PASAT = paced auditory serial addition test, TFESI = transforaminal epidural steroid injection.

**Table 4:** Correlations between microRNA-155 serum level, pain and disability scales, and cognitive function tests in the case group before and 1 month after TFESI.

		MicroRNA-155 serum level before TFESI	
		(*r*) coef.	*p* value
Pain and disability scales before intervention	NRS	0.710	< 0.001⁣^∗^
	ODI	0.408	0.006⁣^∗^
	FRI	0.298	0.050⁣^∗^

Cognitive function tests before intervention	PALT	−0.602	< 0.001⁣^∗^
	COWAT	−0.541	< 0.001⁣^∗^
	PASAT	−0.487	0.001⁣^∗^

		**MicroRNA-155 serum level after TFESI**	
		**(*r*) Coef.**	**p** **value**

Pain and disability scales after intervention	NRS	−0.045	0.771
	ODI	0.074	0.635
	FRI	0.001	0.994

Cognitive function tests after intervention	PALT	0.052	0.737
	COWAT	−0.131	0.395
	PASAT	−0.056	0.717

*Note:* (*r*) Coef.: Pearson correlation coefficient, *p* value ≤ 0.05 is considered significant.

Abbreviations: COWAT = controlled oral word association test, FRI = functional rating index, NRS = numeric rating scale, ODI = Oswestry disability index, PALT = paired associate learning test, PASAT = paced auditory serial addition test, TFESI = transforaminal epidural steroid injection.

## Data Availability

Authors report that the data and materials that support the results or analyses presented in the current study will be freely available upon request.
